# Vesicular lesions in a neonate: what's your diagnosis?

**DOI:** 10.1590/S1679-45082016AI3655

**Published:** 2016

**Authors:** Marta Sofia da Cunha Alves Machado, Elsa Cristina de Freitas Lima Teixeira, Lígia Maria Nogueira Ferreira, Lígia Raquel Gonçalves Basto

**Affiliations:** 1 Centro Hospitalar do Baixo Vouga, Aveiro, Portugal; 2 Centro Hospitalar Tondela-Viseu, Viseu, Portugal; 3 Maternidade Dr. Daniel de Matos, Coimbra, Portugal

Incontinentia pigmenti (IP) is a rare genodermatosis transmitted as an X-linked dominant trait, occurring in 1:50,000 newborns.^(1–3)^ It is frequently lethal in males.^(1–3)^ In this disease, basal cells in the epidermis lose melanin that is collected in the dermis.^(2,3)^ Typically, skin manifestations progress through four stages: vesicular (evident at birth or within the first few postnatal weeks); verrucous; hyperpigmented, and hypopigmented.^(1–3)^ Extracutaneous involvement occurs in 80% of patients.^(1)^ A skin biopsy and/or genetic testing for mutations in NEMO/IKK-gamma confirm the disease.^(1–4)^

A female full-term newborn presented with multiple vesicular lesions in the first 12 hours of life. Given the suspicion of neonatal herpes, she was initiated on acyclovir. Detection of herpes simplex virus by culture and polymerase chain reaction were negative (blood and skin lesions). Within 36 hours, vesicular and hyperpigmented linear skin lesions were apparent, distributed along Blaschko lines, evoking IP. At this time, the mother revealed that she and her older daughter also had some kind of “water bubbles” at birth, information that supported the diagnosis.

**Figure 1:**
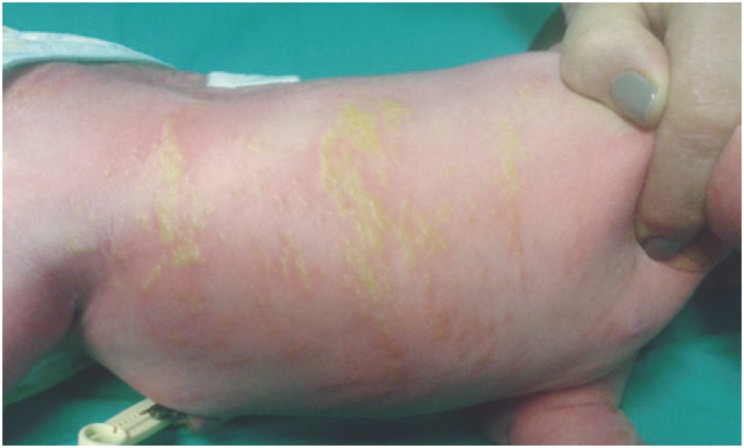
Hyperpigmented linear skin lesions following Blaschko lines

**Figure 2:**
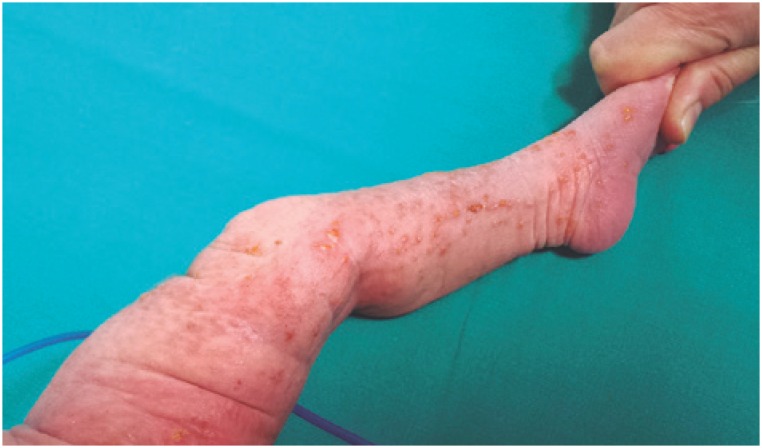
Hyperpigmented linear skin lesions on the leg

In IP, a high level of suspicion is necessary. Differential diagnosis of IP varies according to the stage of the disease.^(1–5)^ In the first stage, it can be confused with herpes simplex, epidermolysis bullosa, or bullous pemphigoid.^(1,5)^ Since neonatal herpes and IP can coexist, early treatment with acyclovir should be started until definitive diagnosis is made.^(1,3)^ The differential diagnosis of verrucous stage is limited and includes linear epidermal nevus.^(1)^ The hyperpigmented stage is IP's hallmark, but may be mistaken for linear and whorled nevoid hypomelanosis.^(1,5)^ The hypopigmented stage can be misdiagnosed as hypomelanosis of Ito or vitiligo.^(1,5)^

The prognosis of IP is generally good, but a periodic assessment by a multidisciplinary team should be made to rule out visual, motor, or intellectual impairment.^(1–3)^ Genetic counseling is of the utmost importance.^(2,3)^
